# Supplementary material for: Are carbon emissions trading and green financial instruments synergistic? -Comprehensive quantitative research based on content analysis
Source: PLoS One. 2024 Mar 7;19(3):e0298601. doi: 10.1371/journal.pone.0298601 (PMC10919601; doi:10.1371/journal.pone.0298601)
Supplement: S1 Data — (ZIP) [file pone.0298601.s001.zip › Data/地级市Z_F工作报告.zip!/╔┬╬≈/░▓┐╡╩╨/└┤╫╘╣1⁄2╓┌║┼í╛┬φ┐╦╩2╛▌═°í┐_2018.docx]

各位代表：

  现在，我代表市人民政府向大会作工作报告，请予审议，并请市政协委员和列席人员提出意见。

  一、2017年经济社会发展情况

  过去一年，在市委的坚强领导下，我们深入贯彻新发展理念，依靠全市人民共同奋斗，较好完成了市人大四届一次会议确定的目标任务。实现生产总值974.7亿元，增长10.5%；财政总收入85亿元，增长8.6%，其中一般预算收入28亿元，同口径增长7.4%；城乡居民收入分别达到28158元和9394元，增长8.5%和9.4%；124个贫困村、7.35万贫困人口脱贫。

  ——经济运行持续向好。主要经济指标快速增长，生产总值、规模工业增加值、固定资产投资、农村居民收入增速和非公经济占比等指标稳居全省前茅。经济运行中有活力、有潜力的积极因素增多，三次产业融合加快，工业支撑能力增强，规模工业增加值完成420.5亿元，增长15.8%；富硒食品产销两旺，实现规模以上产值397亿元，增长28%，“安康富硒茶”地理标志商标成功注册；文化、旅游、健康、电子商务等产业蓬勃发展，社会消费品零售总额增长14.4%，旅游综合收入增长33.5%；非公经济占比58.1%。金融机构存贷款余额分别增长11.9%和13.9%，存贷比54.5%。公路客货运周转量增长10%，新增市场主体2.55万户。

  ——追赶超越步伐稳健。扭住发展不放松，统筹推进生态立市、开放兴市和产业强市，经济增速连续11个季度领跑全省。实施追赶超越季度考核点评和“项目建设年”活动，完成全社会固定资产投资1149.3亿元、增长24.1%。安康机场和安岚、平镇高速，国省干线改造、引汉济渭、洞河水库、钒氮合金二期、天贸物流城二期等重大项目加速推进，南水汽配HS精锻、奥邦智能工厂、普瑞达电梯技改等30个工业项目建成投产。园区成为最具活力的增长极，以安康高新区为引领，各类园区联动，新建标准化厂房42万平方米，新增省级成长梯队企业93户、市级以上农业产业化龙头企业72家、市级农业园区56个，瀛湖国家级旅游服务业标准化试点通过验收。成功创建为国家森林城市，入选中国养老城市排行榜50强，顺利通过国家公共文化服务体系示范区创建资格评审和国家卫生城市复审。

  ——脱贫攻坚有力有效。把脱贫攻坚作为最大政治任务和第一民生工程，坚持精准扶贫、精准脱贫，层层传导责任压力，推动“八个一批”措施落地生根，新社区工厂扶贫模式被全国推广，教育扶贫模式入编中组部干部培训教材。建立“八办两组”协同作战机制，调整充实脱贫攻坚一线“四支队伍”1153个1.3万人。中省市财政专项扶贫资金投入10.7亿元，整合涉农资金22.57亿元，到位各类扶贫贷款95.8亿元，建立金融扶贫风险补偿金2.45亿元，吸纳社会扶贫资金3.6亿元；与人保财险公司签订15亿元支农融资合作协议，首批6个项目落地资金8300万元。建成贫困村基础设施和公共服务项目698个、易地扶贫搬迁安置房6.33万套。

  ——城乡品质稳步提升。深入推进中心城市“湖城一体、疏解江南、重心北移”战略，安康大道、兴安西路、解放路、巴山路等路段改造完成，东坝汉江大桥、1100个公共停车泊位等市政工程建成投用，城东新区、市直机关北迁及泸康大道二期、高井路、长春路、西津公园、汉江大剧院等项目加快推进，香溪公园免费开放。实施棚户区改造1.53万户，货币化安置1.14万户。成立中心城市四办四镇“两违”管控队伍，依法拆除“两违”建筑7.41万平方米。统筹县城和重点镇建设，完成投资24亿元；岚皋、镇坪创建为国家卫生县城，宁陕创建为国家园林县城，平利美丽乡村建设规范上升为陕西省地方标准，长安镇入选中国特色小镇,旬阳县金寨镇荣获“全国文明村镇”称号。国家4A级景区增加到10个，镇坪飞渡峡获评国家水利风景区。治理水土流失852平方公里，绿化造林70万亩，退耕还林17.5万亩；生态红线划定工作全面启动，土壤环境质量达到国家二级标准；汉江水质保护、治污防霾、节能减排成效明显，河长制得到国家水利部肯定，全市地表水质优良率100%，汉江出陕断面水质稳定保持国家Ⅱ类标准，中心城市环境空气质量优良天数316天，单位生产总值能耗下降3.5%。

  ——开放创新激发活力。强力推进以招商引资为主体的开放合作，组织项目与产品推介、文化交流与体育赛事等活动100多场次。强化领导带头招商，聚焦重点区域，力促精准出击，在北京、上海、深圳、杭州、宁波、郑州、常州、佛山、南平、宝鸡等20多个城市开展产品推销和项目推介，与恒大、复星、华为、中兴、阿里巴巴、方圆药业等知名企业达成合作。“共建丝茶之路•同品安康富硒茶”西北省区系列招商推介、富硒食品进西安送健康“五进”活动、海内外侨商投资安康行、南水北调中线城市文化联盟、“鎏金铜蚕”与安康开放发展论坛等活动圆满成功。营商环境满意度位列全省各市（区）第一。津陕对口帮扶、常州-安康扶贫协作不断深化。全年落地招商引资项目280个，到位资金达600亿元，其中外资1563万美元；新增外贸出口企业33户，外贸进出口总额3亿元、增长48.8%。科技创新工作取得新突破，成功举办第二届中国（安康）创新挑战赛、“圆梦安康”创客大赛，新建国家星创天地1个、省级工程技术中心2个、省级星创天地3个、院士工作站3个、创新专家工作站7个，申请发明专利168件，技术交易合同总额超过1亿元，培育科技型企业200家、高新技术企业12家。深化重点领域改革创新，以“互联网+政务服务”为关键点的“放管服”改革取得阶段性成果，村级财务审计受到中省肯定，农村“三变”改革和科技、教育、卫生、文化等领域改革有序推进，盐业体制改革顺利完成。农村信用社全部改制为农商银行，金融聚集区建设进度加快，29家企业在区域性股权交易市场挂牌，华银科技等3户企业在“新三板”挂牌。

  ——民生建设力度加大。完成财政民生支出225亿元，占公共预算支出的82%，八大民生工程扎实推进。坚持以促进创业带动就业，成功创建为省级创业型城市，新增城镇就业2万人，农村劳动力转移就业63.6万人。实施教育优先发展，投入资金60.59亿元，汉滨初中高新校区、江南小学等235个教育项目建成投用；13年免费教育运行稳定，高考二本上线人数首次突破万人关口，岚皋、紫阳达标国家义务教育基本均衡县。积极推进健康安康建设，市妇幼保健院、精神专科医院建成投用，市级公立医院综合改革深入推进，药品采购“两票制”全面实施，汉滨、汉阴、平利、岚皋荣获全国基层中医药工作先进单位。文化建设取得新成果，举办第十七届汉江龙舟文化节，《汉水安康》入选全国优秀纪录片，《汉江船夫曲》荣获省群星奖，参加陕南民歌大赛和省第八届艺术节取得历史最好成绩，市级方志馆建设获全国表彰，新建基层综合文化服务中心505个，培育规上文化企业100家，文化产业增加值突破30亿元。全民健身运动蓬勃开展，举办第十六届全市运动会、半程马拉松、秦岭山地越野等10多项大型体育赛事，在第十三届全运会上实现奖牌零的突破。建成城市社区日间照料中心15个、农村互助幸福院100个，60岁以上老人家庭医生签约服务率60%以上。

  特别值得一提的是，重大活动安保维稳、普法司法、安全生产、应急救援、市场监管、粮食安全、科学普及、残疾人服务、工人权益保障、妇女儿童、红十字会、慈善事业等其他各方面工作也取得了新的成绩，防汛抢险救灾工作受到省防总通报表彰，公众安全感指数连续六年稳居全省前列。

  各位代表！过去一年，在宏观经济环境复杂多变的情况下，面对20年一遇的强秋淋严重灾害，我们紧紧依靠全市人民的智慧和力量，对标忠诚、干净、担当、责任、落实、奉献的工作标准，用敢想敢干、善作善成的责任担当，用苦干实干、拼抢一流的进取精神，赢得稳中快进、持续向好的经济社会发展势头。成绩来之不易，是全市上下共同努力的结果。在此，我代表市人民政府，向全市广大干部群众，向各位人大代表、政协委员、离退休老同志和社会各界人士，向驻安部队、武警消防官兵、政法干警和中省驻安单位，致以衷心的感谢和崇高的敬意!

  总结2017年的工作和成绩，我们尤其要为基层干部职工点赞。在脱贫攻坚主阵地，镇村干部、第一书记、驻村工作队员等万余名干部职工全身心投入，他们用责任、奉献和夜以继日的工作，温暖着每一个贫困群众和村民的心；在抢险救灾最前线，数万名干部职工与灾区群众一起，并肩作战，共度难关，谱写了以人民为中心的实践篇章；在信访维稳、“两违”管控、城镇管理、项目建设征地拆迁和环境保障一线，一大批干部职工默默无闻、攻坚克难，保障了良好的发展环境和重点项目建设进度。这些最美基层干部职工的感人事迹，凝聚了安康加快发展、追赶超越的正能量，激发了埋头苦干、砥砺前行的原动力。实践充分证明，基层干部职工是安康发展稳定的中坚力量，是一支敬业奉献、能打硬仗、可以信赖的队伍。在此，我向广大基层干部职工道一声：辛苦了，向你们致敬！

  在总结成绩的同时，我们也要清醒认识到：我市经济总量小，综合实力弱，数年来较快的经济增速并未能改变欠发达的基本市情。部分行业和企业经营困难，大项目好项目储备不足，动能转换接续不足，经济持续快速增长的基础不牢。生产要素短缺，市场主体不强，产业链短而低端，集群化水平不高，开放发展的深度、广度有待拓展，脱贫攻坚到了最艰难的决战阶段，贫困基数大，脱贫任务重，攻坚难度大。环境保护、公共服务、城市管理、营商环境、诚信建设等方面还有很多短板。一些地方和干部缺乏敢想敢干的闯劲、久久为功的执着、改革创新的魄力，不想为、不会为、不敢为现象依然存在。面对这些困难和问题，我们必须积极作为，切实加以解决。

  二、2018年政府工作的总体要求

  党的十九大确立了新时代的指导思想和基本方略,描绘了我国未来发展的宏伟蓝图。新时代要有新气象，安康定要大作为。2018年政府工作的总体要求是：深入学习贯彻党的十九大精神，以习近平新时代中国特色社会主义思想为指导，按照中央经济工作会议以及省委十三届二次全会、市委四届五次全会的总体部署，聚焦追赶超越目标，落实“五新”战略任务，坚定“发展为要、生态立市、开放兴市、产业强市”实施路径，在紧扣高质量发展的前提下实现高速度增长，突出抓好扩大有效投资、提高供给质量、拓展开放空间、推进乡村振兴和提升脱贫质量、打造“秦巴明珠”、增进民生福祉六大重点工作，为全面建成小康社会、加快建设西北生态经济强市奠定坚实基础。

  经济社会发展的主要预期目标是：生产总值增长9.5%，力争达到10%；全社会固定资产投资增长15%；规上工业增加值增长13.5%，力争达到14%；社会消费品零售总额增长13%；财政总收入和一般预算收入分别增长4%和6%；城乡居民可支配收入分别增长8.5%和9%；完成省定主要污染物减排和节能降耗任务，汉江出境断面水质稳定保持国家Ⅱ类标准；实现镇坪县脱贫摘帽和403个贫困村18万贫困人口脱贫退出。

  做好今年政府工作，首要的是全力满足305万人民群众日益增长的美好生活需要，凝心聚力破解我市发展不充分不平衡的问题。工作中必须牢牢把握以下重点：

  一要扎实践行新发展理念。发展是解决一切问题的基础和关键，加快发展、追赶超越在相当长时期内始终是安康的第一要务。政府工作一刻都不能忽视发展，一刻都不能放松发展。我们要以新发展理念为理论指导和行动指南，崇尚创新、注重协调、倡导绿色、厚植开放、推进共享，着力补短板、强基础、蓄势能，推动经济发展质量变革、效率变革、动力变革，改善发展条件，增强综合实力。

  二要坚持以脱贫攻坚统揽全局。脱贫攻坚是最大的政治任务和发展机遇。我们必须进一步提高政治站位，强化系统思维，坚持精准方略，以产业和就业扶贫为主抓手，统筹“八个一批”脱贫举措，坚持扶贫与扶志扶智相结合，聚焦深度贫困打好“攻坚战”，着眼长远发展打好“持久战”，着力在加快发展中消除贫困、改善民生。

  三要坚定追赶超越实施路径。牢记“四区”叠加和欠发达的基本市情，以时不我待的紧迫感、舍我其谁的责任感，扭住发展不放松，把生态作为立市之本，扩大思想、思路、区域、产业、市场、项目等全方位的开放，加快推进以绿色工业体系为主导的工业化进程，以领先全省的高速度增长保障高质量发展。

  四要提升发展质量和效益。以实现高质量发展引领高速度增长，拉高追赶超越的质量和效益标杆，放大生态资源比较优势，加快构建绿色循环产业体系，实现既有总量上的快速增长、更有质量上的稳步提升。

  五要推动招商引资实现新突破。坚定不移把招商引资作为开放发展第一抓手、追赶超越最强动力，打造一流的营商环境，下硬茬优化环境，下功夫拓展领域，下决心提升层次，开放吸引“外资”，积极争取“央资”，深入挖掘“内资”，充分激活“民资”，创新渠道“融资”，打造内陆开放发展新高地。

  三、强化关键支撑，扩大有效投资

  把抓项目作为稳增长的关键。实施210个市级重点项目，完成年度投资465亿元，着力补齐基础设施、产业发展、生态环保等领域的短板，促进经济持续快速发展。抓实基础设施项目，力促西渝高铁西安至安康段，石泉、汉阴、平利通用机场开工，加快安康机场和平镇、安岚、石宁高速、阳安铁路二线建设，扎实推进750千伏输变电工程、旬阳至桐木高速连接线、汉阴凤凰山隧道项目前期工作，实施月河、平利330千伏输变电工程，加强城乡宽带网络建设和业务发展。抓好重点产业项目，强力推进西北电子信息产业基地、智能终端电子信息产业园、硒谷动力产业园、西北纺织服装产业城、陕西建工新型建材工业园，北医大新药研发及生物医药工程、南水汽配二期和旬阳、白河电站等项目建设，大力促进恒大未来城、颐高新经济产业城、旬阳高新区富硒产业园等项目早开工。继续支持新社区工厂加快发展，扩大在移民搬迁社区的覆盖面，带动群众就地就业增收。抓紧生态环保项目，高标准实施中心城市水环境治理，深入推进大瀛湖生态环境保护治理，加强月河综合整治、蒿坪河流域污染防治与生态保护系统建设，在沿江沿河集镇、社区再建一批污水处理设施。精心谋划接续项目，坚持用创新思维审视现实条件和优势资源，牢牢把握政策走向和市场动向，突出基础设施、富硒产业、包装饮用水、能源环保、装备制造、特色小镇、现代物流、电子商务、公共服务等重点领域，创造性谋划一批高成长性项目，支撑和引领可持续发展。

  以园区承载培育新增长极。把园区作为加快发展的平台和载体，以完善基础设施、提升综合承载能力、促进产业集聚为重点，打造追赶超越增长极。安康高新区要把“创新引领、产业支撑、产城融合”作为主攻方向，突出抓好泰远科技产业园、纳米材料产业园、医疗器械产业园等36个工业项目建设，力促工业产值突破200亿元；实施基础设施、公共服务、人居环境提升工程，建成市一小高新校区、青少年足球训练基地，加快建设天贸物流城二期、生态健康养老城和市民服务中心、鎏金蚕广场、安康中学高新校区、国际标准医养特色医院，建成运营瑞斯丽酒店和北城中央、红星美凯龙等商业综合体，促进人口人才和城市要素加速聚集。恒口试验区要把培育产业、打造副中心作为工作重心，从机制创新和招商引资入手，激发改革发展的动力和活力；强化基础设施配套，新建标准化厂房5万平方米；推进香山小镇、凤栖花谷等田园综合体建设，加强明清古街保护开发；开工建设恒河水库、鸿志学校、仁济医院；建成垃圾处理场。瀛湖旅游区要坚持景区开发与环境保护相结合，加快“水画小镇”建设，启动玉兴岛综合改造，以服务标准化提升旅游接待数量和质量；加强景区资源综合管控与旅游市场整治，清理整顿规范网箱养殖，加快创建国家5A级旅游景区，打造瀛湖旅游新形象。县域工业集中区及飞地经济园区要围绕做大工业总量、提升运行质量，新建25万平方米标准化厂房和一批配套设施，打造10个左右产业集群，加快培育石泉等工业园升级为省级经开区，创建4至5个省级县域工业集中区示范区。现代农业园区要围绕做优产业、打造品牌、提升效益，以生态化、标准化、规模化、循环化为引领，以“千村千园”为抓手，实施产业融合与园区提升工程，新增市级以上现代农业园区60个，培育航母型现代农业园区20个，创建生态循环园区20个，打造三产融合园区10个。

  大力支持创新创业。发挥政府资金撬动作用，激发社会资本活力，组建创业创新基金，以公益参股、贷款风险补偿、股权投资等多种方式，支持各类创业团队、小微企业和成长性企业发展。鼓励市属国有企业在风险可控、安全流动的前提下，参股萌芽期、初创期、成长期等“双创”型企业。以安康高新区为龙头，发挥园区、孵化器、工程技术中心、科技资源统筹中心等科技创新平台的协同效应，结合金融聚集区建设，集聚创业投资团队。加快以企业为主体的技术创新，持续抓好200家科技型企业培育。强化政策支持和环境保障，促进高校毕业生、外出返乡人士创新创业。积极对接各类创新创业活动，让“双创”在安康大地蔚然成风。构建亲清新型政商关系，创新政企互动模式，建立精准支持政策，完善正向激励机制，营造企业家健康成长环境，弘扬企业家精神，凝聚崇尚创新创业正能量。

  四、加快产业融合，提高供给体系质量

  力促工业增量提效。围绕构建绿色循环产业体系，进一步强化工业主导地位和支撑作用。坚持改造提升与引进培育相结合，持续抓好富硒食品、新型材料、装备制造、清洁能源、生物医药、纺织丝绸等绿色工业，延长产业链，提升价值链，保持定力，久久为功。发挥水资源质优量大优势，依托包装饮用水产业联盟，力促与全球全国行业领军企业合作，支持真硒水、天一泉、富硒宝、巴山雪等企业做强，培育茶饮、果酒和功能饮料等关联产业，打造“安康水•健康水•富硒水”品牌。围绕打造区域玩具生产基地的目标，聚力支持和全力吸引全国毛绒玩具等企业在安康落地聚集。加快推进镇坪中药配方颗粒、华秦全钒储能、华润风力发电等项目，强化旬阳烟厂、华银科技、奥邦锻造等40户重点工业企业运行保障，加大政企结对帮扶的力度。全面落实工业稳增长促投资政策措施，打好减税降费、简政放权、银企合作、环境保障“组合拳”，坚定支持民营经济、实体经济发展壮大，培育规上工业企业40户、产值过5亿元企业5户。

  打造特色高效农业。以农业供给侧结构性改革为主线，持续聚焦生猪、魔芋、茶叶、核桃和生态渔业，推动建立产业融资平台，着力打造特色高效现代农业体系。一是育良种，建设陕茶一号、核桃和魔芋良种繁育示范园各20个，生态渔业滤食性品种繁育示范园10个。二是建基地，新建和改造茶园14万亩，实现茶产业综合产值80亿元；创建阳晨生猪产业联盟循环示范园30个，全市生猪出栏稳定在300万头以上；力促魔芋、核桃种植面积达到40万亩、180万亩；围绕渔业养殖与环境保护、旅游发展相辅相成、相得益彰的目标，清理整顿规范瀛湖主景区网箱养殖，发展滤食性鱼类，在秦巴山涧发展特色冷水鱼，水产品保持4万吨以上；严守耕地红线，稳定粮食生产和蔬菜种植，提升蚕桑、烤烟、林果等传统产业；创建省级山林经济示范县2个、示范镇20个。三是抓龙头，实施“十百千万”农业经营主体培育工程，坚持“企业+基地（合作社）+农户”发展模式，新培育茶产业省级龙头企业5家，SC认证企业150家；创建市级以上魔芋加工龙头企业6家。四是创品牌，加强富硒产业基础研究、产品研发、标准制定和品牌建设，通过地方立法严格规范和管控“安康市富硒食品产品专用标志”及“安康富硒”称谓的使用，呵护和提升“中国硒谷”和“安康富硒”的生命力和影响力；推进农业标准化和“三品一标”农产品认证，推进茶叶品牌整合，组建运营富硒茶业集团和秦汉古茶公司，健全“安康富硒茶”地理标志商标使用和保护机制，完成安康富硒猪肉国家行业标准发布，启动安康富硒魔芋、富硒鱼、富硒核桃国家行业标准立项工作，加快紫阳县地标产品省级示范县建设，积极申报石泉蚕桑、镇坪黄连、旬阳拐枣国家地理标志保护产品，推进岚皋魔芋、平利绞股蓝、镇坪中药材和宁陕天麻、猪苓等省级知名品牌示范区创建。

  激活消费市场潜能。围绕建设“秦巴明珠”生态旅游城市，坚定全域旅游发展方向，扎实实施旅游产业发展三年行动计划，推动旅游与其他领域深度融合，深化旅游“厕所革命”。突出抓好游客服务中心建设、4A景区品质提升、瀛湖文旅产业基地、南宫山国家5A景区创建、旅游市场综合整治和生态旅游服务业标准化建设，提升安康旅游发展整体水平，支持石泉、岚皋、宁陕创建国家全域旅游示范县，启动全域旅游示范市创建工作；加强旅游区域协作，打造秦巴山区生态文化旅游区和大巴山风景道，培育健身休闲游、医疗康养游、山地森林游和低空飞行游等新的旅游业态。深入实施“互联网+”行动计划，推进三次产业深度融合、协同发展，形成新的生产方式、产业形态、商业模式和经济增长点；培育电子商务、现代金融、大健康产业和分享经济、消费经济等新型业态，以高质量供给激活市场、扩大消费，力促国家电子商务示范县覆盖全部县区。叫响做靓推广“安康美食”品牌，推动商贸物流、住宿餐饮、文化演艺、交通运输等服务业转型提升。全年新增市场主体3万户以上、力争总量达到20万户。

  五、拓展开放空间，以开放促发展、促改革、促创新

  扩大安康影响。互联网时代，信息产生价值，影响力成为生产力。进一步扩大对外开放，加快发展开放型经济，是追赶超越的必由之路。一方面要厚植比较优势，打造开放合作的核心竞争力；一方面要创新思维和模式，把宣传推介安康贯穿政务、商务和各类节会活动的全过程，不断提升安康知名度、美誉度和吸引力。提升良好生态影响力，积极争创国家生态文明建设示范区，让好山、好水、好空气成为安康的“金字招牌”，让绿色、生态、富硒成为开放发展的核心优势。提升地域文化影响力，大力弘扬安康由来已久的开放包容、兼收并蓄的人文精神，深入挖掘移民文化、水运文化、汉水文化的深远影响和现实意义，打造独具特色、不可复制、国内外知名的“丝路源点•金蚕之乡”靓丽名片。提升营商环境影响力，实施“优化营商环境十大行动”，拿出最好的资源、最好的政策、最好的服务，用最大的诚意识商、招商、安商、亲商、富商，打造全国一流的营商环境，形成产业、投资、人才等资源要素引力场。

  聚力“双招双引”。深入实施开放发展行动计划，聚力招商引资和招才引智，主动融入“一带一路”和长江经济带、汉江经济带建设，紧盯珠三角、长三角、京津冀和关天、成渝、江汉经济圈等热点区域，抓住东中部地区产业转移、东西部扶贫协作和天津、常州对口帮扶等历史机遇，突出产业集群和产业链招商，大力引进装备制造、新型材料、生物医药、富硒食品、纺织丝绸、电子信息、健康养老、生态旅游等产业项目。确定2018年为“营商环境提升年”和“招商引资突破年”，坚持内资与外资、国企与民企、本地企业与外地企业、大项目与中小项目一视同仁，由各级政府和部门“一把手”负总责，采取更大气魄、更新思路、更强力度、更实举措，加强招商政策落地和环境保障，遵循“热情接待、积极促进、支持到位、特事特议、制衡有效”原则，落实市县镇（办）政府主要领导“亲自招商、亲自接待、亲自洽谈、亲自协调”要求，推动一批好项目大项目落地，确保招商到位资金600亿元以上。坚持“招商引资+产品推介”有效模式，强化专业招商、以商招商、产业链招商、领导带队招商和小分队跟进招商，鼓励本地人就地创业和在外创业成功人士回乡投资兴业，建立重点领域委托招商、重点地区驻地招商、重点行业顾问招商机制，建设高水平专业化招商队伍。强化招商引资目标考核和奖惩激励，用好“三项机制”，宽容失误失利，创造放手招商、大胆作为的良好环境。统筹招商引资与招才引智，强化吸引人才人口政策措施，继续实施“百千万人才工程”，积极吸引人口聚集，为追赶超越提供人力资本与人力资源保障。发挥和扩大区位交通优势，着力培育枢纽经济、门户经济和流动经济，积极打造川陕鄂渝“融通枢纽”、陕西向南开放“第一门户”、丹江口库区及上游地区物流中心。建设安康口岸，通畅海关和进出口检验检疫业务。

  深化改革创新。扎实推进全国发展改革试点市建设，突出抓好重点领域改革创新，让创造社会财富的源泉充分涌流。持续推进“放管服”改革，全面实施市场准入负面清单管理，认真落实减税降费措施，坚决斩断伸向企业和项目的一切“黑手”；深化相对集中行政许可权改革，下决心打破互为前置的审批怪圈，实施“减证便民”行动，让群众办事更方便；加快“双随机、一公开”监管全覆盖和综合执法体制改革，建立失信联合惩戒机制，让违法者付出代价。加快公用事业领域改革，鼓励社会资本进入，以竞争推动服务质量提升。深化财税体制改革，完善政银企合作平台，精准对接融资需求，鼓励和支持企业上市融资，对国内A股上市企业给予一次性500万元财政资金奖励。加快高新金融聚集区建设，提升金融再创新和自主创新能力，监督、管理和服务属地的全部金融活动，防范和化解金融风险。推进生态环保领域改革创新，打造“绿水青山就是金山银山”的“安康模式”。加快统计管理体制、公立医院、城市管理执法体制和文化市场综合执法等改革，力促科技、教育、司法、社会治理等重点领域改革取得实效。统筹抓好国家主体功能区建设试点示范、政策性金融扶贫实验示范、低碳城市试点、医养结合试点和全省山林经济示范市、生态养老试点市、镇级小城市综合改革试验区建设，积极探索，大胆实践，确保规定动作到位、自选动作出彩，力促改革出成效、创新出经验。

  六、推进乡村振兴，提升脱贫攻坚质量水平

  坚决打好脱贫攻坚的关键之战。实施乡村振兴、决胜全面小康，摆脱贫困是底线任务。必须坚持以脱贫攻坚统揽全局，层层夯实责任，强化精准施策，由注重外部帮扶向注重外部帮扶与激发内生动力并重转变，由基础设施建设为主向产业就业转变，由大规模搬迁建房为主向搬迁后社区配套和新社区建设转变，走出一条符合安康实际、具有安康特色的减贫之路，打好决胜深度贫困的关键之战。统筹近期脱贫与长远发展，把培育产业作为根本之策，全面推行“企业+基地（合作社）+贫困户”模式，大力培育新型农业经营主体和技能型农民，带动贫困群众增收致富。强化保障性扶贫，用好政策机遇，着力补齐基础设施和公共服务短板。2018年全面完成后三年的扶贫搬迁任务，推广“社区+工厂”“社区+景区”“社区+园区”等就业模式，因户因人解决好搬迁群众就业问题。基础设施和公共服务建设“三年任务两年完成”，重点抓好当年计划脱贫退出的村，突出抓好深度贫困县和贫困村，确保道路、饮水、用电等基础设施和安全住房按期建成达标。扎实推进东西部扶贫协作、中省定点扶贫和科技扶贫，持续推进“五大工程”，深入实施“百企帮百村”“青年助力脱贫攻坚”等社会扶贫活动，凝聚决胜脱贫攻坚的有效合力。坚决纠正和防范检查多、填表多、会议多、政策多变等问题，力戒形式主义、官僚主义和急躁浮躁风。

  充分激发农业农村发展活力。认真落实中央农村工作会议提出的乡村振兴任务书、路线图和时间表，按照产业兴旺、生态宜居、乡风文明、治理有效、生活富裕的总要求，制定乡村振兴发展规划，着力构建现代农业产业体系，加快建设美丽宜居乡村，逐步实现城乡一体、融合发展。积极稳妥推进农村土地制度和集体产权制度改革，深化“两权”抵押贷款改革试点，加快“资源变资产、资金变股金、农民变股东”，促进农户与经营主体“联产联业联利联心”，推动建立农民分享产业链收益的长效机制，多渠道增加农民收益。推行村级组织+涉农企业（合作社）+“三变”的基本方式，优化农村资源、资产、资金等要素配置，鼓励工商资本、民间资本进入，形成规模集聚效应，促进农业和农产品加工业发展，激活“三农”发展内生动力。落实粮食安全责任制和“菜篮子”市县(区)长负责制，保障区域粮食安全。依托企业和新型农业经营主体，加强职业农民和就业创业技能培训，推进农产品多元开发和精深加工，培育观光农业、美丽乡村、农村电商等新型业态。加强和创新农村基层社会治理，深入推进“诚孝俭勤和”新民风建设，健全自治、法治、德治相结合的治理体系。

  全面加强农村基础设施建设。实施乡村振兴战略，基础建设必须先行。围绕推进城乡发展一体化，强化农村基础设施的公共产品定位，加大政策投入力度，创新投融资模式和运行方式，推动农村基础设施条件显著改善。以道路、水利、电力、环保、通讯、宽带网络为重点，加大扩面提等项目争取及资金投放力度，推进城乡基础设施互联互通、共建共享。按照“四好农村路”要求，实施农村交通攻坚行动，两年内新建和改造农村公路8000公里。加快水毁基础设施修复重建，抓好农村环境与中小河流治理工程，实施农村通组硬化路、安全饮水、自然村动力电“三提升”工程，大力推进“厕所革命”和农业面源污染、村容村貌治理。积极拓展农村宽带网络应用领域，提高相关业务性价比，为农村发展“互联网+”提供有力支撑。

  七、突出龙头带动，打造休闲养生“秦巴明珠”

  加快建设区域中心城市。在秦巴区域大格局中谋划中心城市建设，坚定“湖城一体、疏解江南、重心北移”战略不动摇。依法依规启动行政中心北迁工作，继续实施市级机关北迁和江南老城区“拆公还公”“减容建设”，强力推进九大片区棚户区改造，注重历史建筑保护与利用，留住安康人的“老城记忆”。坚持生态优先，突出规划引领，强化市区一体，统筹推进城东新区建设。高标准实施“中心城市一江两岸亮化美化工程”，完成泸康大道二期和巴山路、江北大道综合改造，加快长春路、北环线、机场干线、新时代大道建设进度，加快推进南环中路、育才东路、瀛湖路改造和南平路、董家沟路建设，规划启动江南城区老街道文化和功能提升工程，完善内畅外联的城市路网体系。规划建设安康高新区医养新城项目，建成汉江大剧院、工人文化宫。完成江南污水处理厂迁建。按照信息共享、以点带面的原则，从民生领域入手启动“智慧安康”建设试点。巩固国家卫生城市、国家森林城市创建成果，统筹推进环保模范城市、文明城市、综治优秀城市“五城联创”，强化城市管理协同联动和综合执法，持续抓好精品示范街区建设，提升安康文明开放新形象。

  着力打造有活力的小城镇。坚持城乡一体化发展方向，按照县城有活力、小镇有特色、社区和而美的要求，强化规划引领和项目支撑，力促城乡建设与管理融合，推动基础设施向乡镇延伸、公共服务向农村覆盖。启动月河快速干道建设，实施月河区域联动和综合整治，带动省主体功能区规划中我市月河川道“重点开发区域”和城镇带发展。创新体制机制，加快恒口镇级市建设，探索城乡统筹一体化发展经验。支持9县城开展各层级的城市创建活动，以创促建提升城镇化水平。建成79个城镇新型智慧社区，改善居民生活环境。扎实推进平利长安、石泉池河、宁陕皇冠、旬阳蜀河等特色小（城）镇建设，继续抓好16个重点镇、3个省级文化旅游名镇（街区），推进农村新型社区建设，让城乡居民有更多获得感。

  全面建设生态宜居美丽安康。深化国家主体功能区建设试点示范，以厚植生态优势、提升环境质量为目标，认真落实环境保护“党政同责、一岗双责”和政府属地管理责任、环保部门监管责任、企业主体责任，全面完成中省环保督察反馈问题整改，高质量完成第二次全国污染源普查。深入实施汉江水质保护“十大专项行动”，完善“河长+警长+督察长”管理责任体系，高标准实施沿江沿河城镇“两厂(场)”建设，加强瀛湖湖泊综合治理，推行重点监控源第三方管理运营。突出危化品运输、尾矿库、农业面源污染等重点，严格落实防控责任和应急措施，推行危化品道路运输“入境检查、重点护送、出境通报”工作机制，确保不发生重大环境污染事故。坚决打好“蓝天保卫战”，持之以恒开展秸秆焚烧、城市扬尘等专项整治，环境空气质量优良天数保持在310天以上。继续实施造林绿化和土壤污染治理、山水林田湖草生态修复。完成166个村农村环境综合整治任务。强化生态红线管制，加强秦岭生态环境保护和修复治理。支持企业实施清洁化生产、循环化改造，加强对固体废物与垃圾回收处理的监管，开工建设中心城市垃圾焚烧发电项目。全面开展国家低碳城市试点，倡导绿色环保生活方式，积极推广使用新能源，开展节约型机关、绿色单位创建等活动，引导全社会共建绿色家园，打造优美宜人的生态空间。

  八、坚持为民惠民，在发展中增进民生福祉

  完善公共服务。围绕教育优先发展，以提高教育质量为主攻目标，加快推进第三期学前教育行动计划、义务教育学校标准化建设、高中教育普及攻坚计划，实施中心城区教育提升工程，探索集团化办学模式与运行机制，集中力量支持汉滨区创建国家义务教育基本均衡县（区）；支持安康学院建设有特色、高品质的一流学院，以安康职业技术学院为龙头，深化产教融合与校企合作，着力培养技能型实用人才。深化医疗卫生综合改革，抓好医联体建设和远程诊疗，推动优质医疗资源下沉，加快形成分级诊疗格局；建成市公共卫生服务中心、市眼科医院和市人民医院门诊综合楼、市中心医院全科医生培训基地，提升县级医院服务能力，推进镇卫生院、村（社区）卫生室标准化改造；抓好市中医院国家中医传承创新能力建设项目，加快建设秦巴区域中医诊疗中心；推进市中心医院江北院区扩能提升和江南院区内涵式改造，完成汉滨区一院、三院建设，加快平利县医院二期、旬阳县中医院迁建和宁陕县中医院建设；扎实推进国家医养结合试点市建设，发挥市场主体作用，政府积极作为，推动医养结合与生态健康养老产业发展。务实推进国家公共文化服务体系示范区创建，启动安康美术馆、香溪书院和县区“三馆一院一中心”等一批公共文化设施项目，实施“十强百优”文化人才扶持培养计划，持续推进汉剧振兴工程，加强地域文化研究和文化遗产保护利用，大力培育文化演艺、广播影视、工艺美术、动漫游戏等文化产业。深入实施全民健身运动，以安康龙舟赛、半程马拉松、秦岭山地越野挑战赛等为重点，全年组织各类体育赛事50场次以上；扎实推进市体育运动中心前期工作，加快建设汉江全民健身长廊，高标准实施140个体育惠民工程，推动县城公共体育场全覆盖。实施社会保障扩面提标，建设市生态休闲养老服务中心和10个居家养老服务示范社区。完善城乡社会救助和社会福利体系，关爱农村留守群体和社会困难群体，让安康大地充满大爱与温暖。

  加强和创新社会治理。按照中省部署，深入扎实开展扫黑除恶专项斗争；实施“雪亮工程”，强化“三个精准防范”，打造立体化、信息化的社会治安防控体系，推进“智慧警务”；坚持民生警务理念，深化“破小案、暖民心”活动，大力整治“盗抢骗”“黄赌毒”等违法犯罪，持续提高人民群众安全感。积极推进安全生产领域改革创新，常态化开展隐患大排查大整治，加快实施尾矿库治理工程，全面建立安全生产双重预防机制，确保安全生产形势稳定。全面推进“七五”普法，加快社会法治进程，推进社会公共法律服务平台建设，完善人民调解、行政调解、司法调解有机衔接、协调联动、高效便捷的矛盾纠纷化解机制，扎实推进“阳光信访”“责任信访”“法治信访”，依法规范信访秩序，预防和化解社会矛盾。全面加强市场监管，聚焦食品药品和产品质量安全，严格事中事后监管，切实管住、管好，支持争创省级质量强县、食品安全示范县和知名品牌示范区。完善社会信用系统，加强社会信用管理和应用，建设诚信社会。加强自然灾害、事故灾难、公共卫生事件和社会安全事件防范与应急处置，对重点企业、重点部位、重大危险源实时监控，切实维护人民群众生命和财产安全。

  各位代表！顺应人民群众新期待和社会发展新要求，今年重点实施“为民惠民八项行动”：

  一是创业就业行动。全面落实新一轮就业补贴和创业扶持政策，积极支持高校毕业生、在外务工返乡人员、本地人等各类群体在安康创业就业。大力发展新社区工厂，积极承接毛绒玩具等产业转移，力争新培育社区工厂100家，吸纳就业1万人。组织免费技能培训3万人次，发放创业担保贷款4亿元，城镇新增就业2万人，城镇登记失业率控制在3.9%以内。

  二是教育提升行动。实施教育项目189个，完成投资5.3亿元，进一步提升城乡基础教育学校办学条件；组织中小学、幼儿园教师参加市级以上培训1万人次；推进中心城区教育提升工程，新增学位5000个，开展集团化办学试点，尽最大努力解决择校热、大班额问题。

  三是便捷医疗行动。扩大“居民健康卡”覆盖面，促进医疗服务全程大提速；实施新生儿52种疾病免费筛查；全市三级以上医院全部开通电话或网上预约挂号，推行同级医疗机构检查、检验结果互认，有效降低患者就诊费用；深入推行分级诊疗和双向转诊，家庭医生签约服务覆盖30%以上的家庭。

  四是安居保障行动。认真组织实施政府购买棚改服务，完成11153户保障性安居工程建设（城市棚户区改造），新增租赁补贴发放2885户，完成农村危改9791户；围绕促进房地产市场平稳健康发展，积极探索建立符合安康市情的多主体供应、多渠道保障、租购并举的住房制度，满足不同层次住房需求。

  五是食品安全行动。按照“四个最严”的要求，以“小餐饮、小作坊、小摊贩”为重点，开展餐饮行业专项整治，加强网络订餐和外卖食品监管，拒绝餐桌污染，确保食品安全，让老百姓吃得放心；新建和改造农贸市场2个、社区便民商业服务中心3个，评选一批市级食品安全示范单位。

  六是公共文体行动。推动安康学院院地共建图书馆尽快向市民开放，中心城区新增24小时无人值守自助书屋20个，促进全民阅读，建设书香安康，提升城市品质；举办群众性公共文化活动50场次以上；实施10个县级公共体育场馆设施“三个一”改造，市县镇村四级学校体育运动设施全部面向社会免费开放。

  七是交通治理行动。加强中心城市道路交通优化和早晚高峰交通疏导，有效消解南北通道、重点路段、校园周边等交通堵点；严厉打击车辆违法营运、乱停乱放行为，规范摩托车、电动车、代步车管理；新增和更新出租车270辆，新增公共停车泊位2000个。

  八是政务便民行动。织密全市政务服务“一张网”，延伸12345便民服务“一条线”，完善公共资源交易“一个平台”， 让信息“多跑路”、群众“少跑路”；推行马上办、网上办、一次办，推出更多“只跑一次”和“不见面”服务事项，办事时限再压缩20%，12345办理不超过5个工作日，降低企业参与公共资源交易成本。

  九、不断提升政府施政能力和服务水平

  新时代赋予新使命，新使命呼唤新作为。重任千钧惟担当，打铁必须自身硬。天道酬勤，幸福都是奋斗出来的。我们一定要以永远在路上的坚韧，锲而不舍地抓好政府自身建设，不断提升施政能力和服务水平，更好地为群众服务、让人民满意。

  一要着力提升“四个定力”。始终把党的纪律和规矩挺在前面，深入开展“不忘初心、牢记使命”主题教育，自觉用习近平新时代中国特色社会主义思想武装头脑，对标政治坚定、纪律严明、本领高强的要求，不断增强“四个定力”，永葆为民务实清廉本色。增强政治定力，旗帜鲜明讲政治，树牢“四个意识”，坚决维护以习近平总书记为核心的党中央权威和集中统一领导，全面贯彻中、省和市委决策部署，做政治上的明白人。增强纪律定力，牢记纪在法前、纪严于法，严格落实中央八项规定实施细则精神和省委、市委贯彻意见，始终心存敬畏、手握戒尺，做到心中有戒、慎独慎微，严于自律，坚守底线，不逾红线。增强道德定力，自觉践行社会主义核心价值观，带头落实“诚孝俭勤和”新民风建设要求，发挥导向和引领作用，以道德力量赢得人民群众、赢得事业成就。增强抵腐定力，把廉洁自律作为关键防线，强化自我约束，防止权力滥用；认真履行廉政建设主体责任和“一岗双责”，更加注重从源头上防控廉政风险，除腐败、零容忍，拒腐蚀、永不沾。

  二要着力提升依法履职能力。政府系统各级干部都要带头尊法学法守法用法，用心谋事、激情干事、善于成事，全面履行经济调节、市场监管、社会治理和公共服务等法定职责，自觉接受人大的工作监督、依法监督和政协的民主监督、媒体的舆论监督、群众的社会监督。完善依法行政体制机制，推进重大决策、政务公开、行政程序法治化，用严密的程序管好权力运行。认真办理人大代表建议和政协委员提案，深化政府信息公开，以公开为常态，不公开为例外，健全和完善政府权力清单、责任清单、负面清单制度，强化对缺位、越位、错位行政行为的监督制约与责任追究，让公共权力在阳光下运行。

  三要着力提升改革创新能力。发挥政府推动改革创新的引领作用，向开放要潜力，向改革要动力，向创新要活力。围绕转变政府职能，扩大“互联网+政务服务”“一站式服务”和公共资源“阳光交易”覆盖面，降低制度性交易成本，增强发展内生动力。推进体制机制创新，营造激励体制机制创新的政策环境和制度环境，充分激发全社会创新活力和创造潜能。紧扣基本公共服务和重大基础设施两大重点，深入推进政府和社会资本合作，多渠道提升共建能力和共享水平。深化事业单位改革，强化公益属性，推进政事分开、事企分开、管办分离，提高社会服务体系建设水平和供给效率。

  四要着力提升执行力和公信力。突出实干为先、落实为要、诚信为本，勇于挑最重的担子、敢于啃最硬的骨头、善于破最坚的堡垒，强化“踏石留印、抓铁有痕”的韧劲，完善一级抓一级、层层抓落实的大督查格局，推行工作任务清单式管理，细化分解到项目、岗位和个人。坚持重点工作公开承诺、跟踪督办、问效问责制度，完善重点项目和重点企业领导包抓、部门帮扶等有效工作机制。围绕提高执行力，倡导雷厉风行、马上就办，说到做到、办就办好，困难不回避、矛盾不上交、工作不拖拉，有决策就有落实、有安排就有反馈、有部署就有结果，确保政令畅通、令行禁止。把诚信施政作为重要准则，以“徙木立信”的态度取信于民，以政府诚信带动市场诚信、企业诚信和个人诚信，营造诚信守法、公平竞争的市场环境，加快建设诚信安康，促进全社会诚信意识和诚信水平提高。

  各位代表！中国特色社会主义进入新时代，安康“追赶超越、绿色崛起”迈向新征程，让我们紧密团结在以习近平同志为核心的党中央周围，在省委、省政府和市委的坚强领导下，不驰于空想，不骛于虚声，携手并肩，奋勇拼搏，共建“秦巴明珠”，共筑美好明天，为全面建成小康社会、加快建设西北生态经济强市不懈奋斗！
